# Valproate-Associated Transaminitis and Rhabdomyolysis

**DOI:** 10.7759/cureus.38348

**Published:** 2023-04-30

**Authors:** Anupam Sharma, Jeannlis Sanchez, Madison Dew, Gursimran Shergill

**Affiliations:** 1 Internal Medicine, Crozer-Chester Medical Center, Chester, USA; 2 Internal Medicine, Jefferson Lansdale Hospital, Lansdale, USA; 3 Internal Medicine, Drexel College of Medicine, Philadelphia, USA

**Keywords:** valproic acid, rhabdomyolysis, transaminitis, seizure medications, rare side effect, acute hepatotoxicity, non-traumatic rhabdomyolysis, valproic acid toxicity

## Abstract

Valproic acid (VPA), a common anti-epileptic with prevalent use, has many side effects such as alopecia, abdominal discomfort, thrombocytopenia, etc. Other than those documented, publications cite the drug's rare side effects, such as hepatotoxicity, coagulation disorders, hyperammonemic encephalopathy, rhabdomyolysis, etc. We present the case of a 24-year-old man who was started on valproic acid after a seizure episode and developed mild transaminitis and rhabdomyolysis within 24 hours of drug initiation. Cessation of the drug led to the resolution of raised creatinine kinase and transaminase levels.

## Introduction

Valproic acid (VPA) is a branched-chain fatty acid that potentiates the effects of gamma-aminobutyric acid-ergic (GABAergic) inhibition on the central nervous system (CNS) as well as suppresses N-methyl-D-aspartate (NMDA)-evoked depolarizations. Its use has become prevalent in conditions such as seizures, bipolar disorder, and migraines. Apart from the common side effects like gastrointestinal upset, fatigue, hair thinning, hypersensitivity reactions, and others [1], some rare side effects of valproate have been published. These include hepatotoxicity, coagulation disorders, pancreatitis, hyperammonemic encephalopathy, thrombocytopenia, and rhabdomyolysis. Hepatotoxicity caused by VPA is a rare occurrence (one in 10,000 patients using VPA), manifesting within weeks to months of initiating drug therapy. However, it is imperative to note that there are some instances where the risk of fatal hepatotoxicity is significantly increased. Severe hepatotoxicity has a higher occurrence in those with a familial predilection and children under the age of three, especially those on multiple antiepileptics and those with signs of developmental delay [2,3]. Additionally, patients with Alpers-Huttenlocher syndrome, a neurometabolic disorder caused by mutations of the mitochondrial DNA polymerase-gamma (POLG), are at higher risk of developing fatal VPA neurotoxicity. If using VPA in these sub-groups, great caution should be taken [4]. Rhabdomyolysis is characterized by the necrosis of muscle and the release of intramuscular contents. It is characterized by myoglobinuria, which leads to acute kidney injury due to prerenal and renal mechanisms [5]. Among the intramuscular contents, there is also creatine kinase (CK), an enzyme essential for buffering and regeneration of adenosine triphosphate (ATP) [6]. Its serum level is the most sensitive indicator of muscle damage and is used to diagnose and evaluate the course of muscle disease [7].

Here we present a rare case of acute mild transaminitis and rhabdomyolysis occurring within 24 hours of initiating VPA.

## Case presentation

A 24-year-old man with a history of intellectual disability and one seizure episode a month ago presented to the emergency room (ER) after a generalized tonic-clonic seizure (GTCS) episode and postictal confusion. He was witnessed having a GTCS in the ER, which was managed with intravenous (IV) benzodiazepines and a load of levetiracetam (LEV) 1 g IV. He had been non-compliant with the home dose of LEV due to excessive sedation for the last two weeks. The patient was lethargic and sedated on physical examination and had significant polyuria. Considering his history of excessive free water intake, serum sodium of 120 mmol/L, serum osmolality of 253 mOsm/L, urine sodium of 11 mmol/L, urine chloride <15 mmol/L, and urine osmolality of 37 mOsm/L, he was diagnosed with psychogenic polydipsia. No acute events were noted on non-contrast computed tomography of the head. Raised creatine kinase (2836 U/L), lactic acid (4.5 mmol/L) levels, and mild elevation of aspartate aminotransferase (AST) (75 U/L) were other significant positives on initial serum analysis. Due to side effects from LEV, he was started on divalproex sodium 500 mg twice a day orally. Hyponatremia was gradually corrected with two doses of desmopressin (2 mcg IV) and six liters of IV dextrose 5% in water in the first 48 hours to correct his sodium to 135 mmol/L. Repeat CK tests done on day two showed an increase to 5,566 U/L with persistent elevation for the next six days. Similarly, liver transaminases had a mild upward trend, raising alanine aminotransferase (ALT) to 64 U/L and aspartate aminotransferase (AST) to 105 U/L. No seizure episodes or muscular traumas were reported during this period. He was noted to have dark urine, but creatinine levels were stable below 1 mg/dL. His divalproex was held after a dose on the evening of day six, and the CK test measured on the morning of day eight was 1,642 U/L. Similarly, liver transaminases were on a downward trend, with ALT at 55 and AST at 95. He was discharged with a prescription for lacosamide 100 mg twice daily and a recommendation to limit free water intake and substitute it with electrolyte-rich drinks.

## Discussion

Sodium valproate-induced rhabdomyolysis is a relatively rare and poorly documented potential side effect. Interestingly, the majority of the cases documented presented with muscle weakness after months of usage, with one case report showing muscle weakness after a few days [8]. Our case presented asymptomatically, with rising creatinine kinase as its only manifestation after just one day of usage. Based on these other case reports, muscle weakness likely would have developed, but rising CK levels were identified early, and valproate was discontinued before any symptoms developed.

Valproate is a broad-spectrum antiepileptic used to treat many seizure disorders. It can also be used in the treatment of diabetic neuropathy, migraines, migraine prophylaxis, suppression of tumor growth, and as a mood stabilizer [8,9]. Unfortunately, it also has its fair share of side effects [8]. Prior research suggests that some of these side effects are due to valproate’s depletion of carnitine stores [9]. Other case reports of valproate-induced myopathy found low carnitine levels, giving us a possible mechanism of valproate-induced myopathy [5]. Carnitine levels were not measured in our patient, as the myopathy was identified and reversed early.

In general, muscle tissue has high blood flow, mass, and mitochondrial energy metabolism, making it particularly susceptible to drug-related damage [8]. Drug-induced myopathy can range from mild weakness to complete paralysis and rhabdomyolysis with acute kidney failure. The little evidence we have so far indicates that valproate-induced myopathy tends to present as proximal lower extremity muscle weakness [8].

Research shows that with any drug-induced myopathy, cessation of the offending agent should ensue at the first indication of myopathy. If stopped early enough, any muscle damage may be completely reversed [8,9]. The start and stop dates of any present or past drugs should be documented. If muscle damage has occurred before the discontinuation of the drug, treatment should first consist of the correction of any metabolic derangements. Muscle necrosis should be controlled by preventing muscle contraction. Absorption of the drug can be hindered by administering activated charcoal, or excretion can be enhanced via diuresis or hemodialysis [10,11]. In our case, cessation of the drug resulted in a decrease in CK levels one day after discontinuation. Should valproate-induced myopathy occur, alternative pharmacological therapies to valproate include lacosamide, lamotrigine, topiramate, and levetiracetam [12].

## Conclusions

Our case is of clinical importance, as not many cases of valproate-induced myopathy have been documented. In our case, myopathy was identified early enough via measurement of CK levels, and cessation of valproate occurred before any symptoms appeared. This raises the question of whether CK levels should be measured after the initiation of valproate to screen for drug-induced myopathy so that an alternative therapy can be started before muscle damage occurs. The significant morbidity and disability that drug-induced myopathy can cause, as well as the fact that early intervention has the potential to reverse the myopathy, make it of vital concern to identify it early and treat it promptly.
